# New Nodule Type Found in the Lungs of *Pomacea canaliculata*, an Intermediate Host of *Angiostrongylus cantonensis*

**Published:** 2018

**Authors:** Yue GUO, Hong Chang ZHOU, Ying DONG, Ting ZHANG, Yu Yang SUN, Jian Feng ZHONG, Yu Liang CAO, Sheng Wen SHAO, Yong Liang PAN, Hai Yan DONG

**Affiliations:** 1. School of Medicine, Huzhou University, Huzhou, China; 2. Infectious Diseases Dept., Huzhou Central Hospital, Huzhou, China; 3. Intensive Medicine, No.98 Hospital of PLA, Huzhou, Zhejiang, China

**Keywords:** *Pomacea canaliculata*, Lung nodule, 18S ribosomal RNA, *Poterioochromonas* sp. *Angiostrongylus cantonensis*

## Abstract

**Background:**

*Pomacea canaliculata* (*P.canaliculata*) lung nodules, were commonly caused by *Angiostrongylus cantonensis* infection. Here, we found a new nodule type without any parasites.

**Methods:**

Overall, 447 P. canaliculata snails were collected in Ning Bo, Zhe Jiang, China in 2018. In order to exhibit the similarities and differences between two nodules types (2018, Huzhou Zhejiang, China), both types were collected in formalin for tissue pathological sectioning. Besides, to obtain the microbial community of the new nodule, the 18S ribosomal RNA (rRNA) gene of it was amplified and analyzed using the Illumina second-generation sequencing platform.

**Results:**

Although two nodules were found in the lungs of *P. canaliculata*, they were different in shape and pathology. Illumina sequencing indicated *Poterioochromonas* sp., a species of golden algae, might be the causing agent of the new nodule.

**Conclusion:**

We firstly found a new pathological nodule type in the lungs of *P. canaliculata*, and this nodule might be induced by golden algae infection, however, the direct link between the golden algae and the new nodules, as well as the nodules’ impact on the snails’ physiology and *A. cantonensis* infection require further study.

## Introduction

The freshwater snail, *Pomacea canaliculata* is a globally invasive species (1). Widely distributed in southeast China, this snail has a bad reputation as the intermediate host of *Angiostrongylus cantonensis*, the causative agent of eosinophilic meningitis (2). In China, *P. canaliculata* is frequently mistaken for *Cipangopaludina chinensis Gray*, a local edible snail and not a natural vector for *A. cantonensis*. This mistake directly induces many cases of eosinophilic meningitis in China.

*Angiostrongylus cantonensis* infection occurs in many *P. canaliculata* organs, including the foot and lung sac (3). *A. cantonensis* infection can leave pathological nodules in the snail host’s lungs. Microscopic detection of larvae nodules in the lungs of *A. cantonensis-*positive snails is the primary pathological sign of *A. cantonensis* infection. These signs are used to characterize *A. cantonensis* infections because it is cheap and efficient (4). Here, we found a new nodule type without any parasites.

## Materials and Methods

### Snail samples and nodule collection

Overall, 447 *P. canaliculata* snails were collected in Ning Bo, Zhe Jiang, China in 2018. After the shell was broken, the snail’s mantle skirt was cut from the body and its lung sac was cut open and flattened in 0.6% physiological saline under a microscope. The prevalence of both the *A. cantonensis*-induced nodules and the new nodules was recorded. Lung sacs with *A. cantonensis*-induced nodules and new nodules were collected in formalin and used for tissue sectioning, followed by hematoxylin and eosin (H&E) staining. Photographs were taken microscopically. The new nodule surfaces were then microscopically cut with a scalpel, gently squeezed with eye forceps and internal substances were collected in 0.6% physiological saline for sequencing.

### DNA extraction and amplification

DNA from the new nodules was extracted using the Magic Mag Micro Genomic DNA Extraction Kit (NO. B518749, Sangon Biotech Co., Ltd. Shanghai, China). Both bacterial 16S rRNA and eukaryotic 18S rRNA were amplified by PCR and sequenced by the Illumina MiSeq platform (Origin-gene Biomedical Technology Co., Ltd., Shanghai, China). The primer information is listed in Table 1; the first primer was used to amplify the bacterial 16S rRNA gene (5–7), and the second was used to amplify the V4 region of the eukaryotic 18S rRNA gene (8, 9).

**Table 1:** Primer Information

***Primers***		***Primer Sequences***
Primer 1	341F	5′-CCTAYGGGRBGCASCAG-3′
	806R	5′-GGACTACNNGGGTATCTAAT-3′
Primer 2	TAReuk454FWD1	5′-CCAGCASCYGCGGTAATTCC-3′
	TAReukREV3	5′-ACTTTCGTTCTTGATYRA-3′

A TransStart FastPfu DNA Polymerase 20-μl reaction system was used in the ABI Gene-Amp® 9700, including 4 μl of 5×FastPfu Buffer, 2 μl of 2.5 mM dNTPs, 0.8μl of forwarding primer(5 μM), 0.8μl of reverse primer(5 μM), 0.4μl of FastPfu Polymerase, and 10ng of template DNA. PCR parameters were as follows: an initial denaturation step at 95 °C for 5 min, 27 cycles at 95 °C for 30 sec, 55 °C for 30 sec and 72 °C for 45 sec, with a final extension step at 72 °C for 10 min.

PCR products were detected by 0.2% aga-rose gel electrophoresis and purified by the AxyPrep DNA Gel Extraction Kit (AXYGEN Biosciences). Quanti Fluor™-ST was then used to quantify the PCR products, and DNA was pooled to construct an Illumina library after Illumina PE250 sequencing (10–12).

### Data and taxonomic analyses

Raw data provided by the PE reads from the Illumina PE 250 were stitched together by the following software: Trimmomatic (13), FLASH (14), Usearch (15), and QIIME (16). The raw data were denoised, trimmed, quality-filtered, and aligned to construct the operational taxonomic unit (OTU) matrix. OTU information was then used to classify groups of closely related individuals.

To reveal the community composition per sample, representative OUT sequences with similar levels above 97% were taxonomically analyzed by RDP Classifier, Bayesian algorithm. QIIME and RDP Classifier (ver. 2.2) were applied for taxonomic research (17–19).

## Results

### Pathological description

**Fig. 1:**
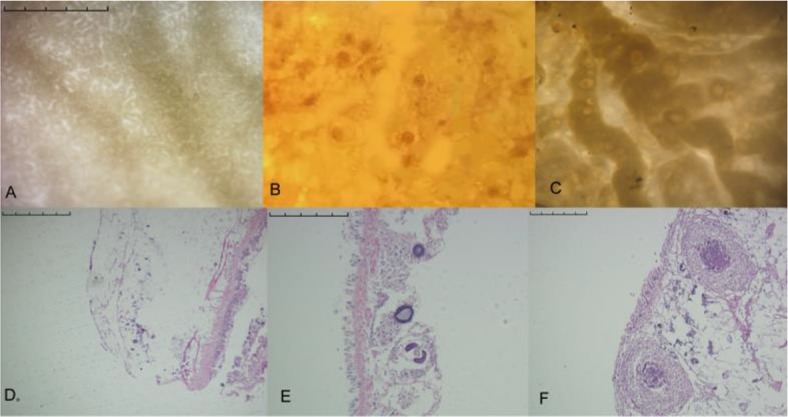
Lung of *Pomacea canaliculata* (*P. canaliculata*). A: Healthy lung with no parasitic infection or other pathological changes; B: Lung with *A. cantonensis*-induced nodules; C: Lung with the new nodules; D: Cross-section of a healthy lung stained with hematoxylin and eosin (H&E), bar=1 mm.; E: *A. cantonensis*-induced nodules, H&E stained, bar=0.5 mm; F: the new nodules, H&E stained, bar=0.5 mm

Nodules in the *P. canaliculata* snail lung sac included two types: *A. cantonensis*-induced nodules and the newly discovered nodules (Fig. 1).

The *A. cantonensis*-induced nodules contained 2^nd^- or 3^rd^-stage *A. cantonensis* larvae. When the nodule was torn open, *A. cantonensis* larvae could be detected microscopically.

The new nodules were the latest discovery, first exhibited here. These nodules were shaped like poached eggs, with two different-sized globes set together. The smaller globe was composed of cells and the larger globe was transparent, surrounding the smaller one to form a poached-egg shape microscopically. The new nodules differed in size, with the smallest being 0.1 mm in diameter, and the biggest being 2 mm in diameter. Both microscopic detection and tissue sectioning showed that these nodules were not parasite larvae (Fig. 1).

Size differences among the new nodules suggested that they were developing and might be caused by micro-organisms infection or cancerous tissues. The new nodules were either masses of exogenous cells or snail cells (Table 2).

**Table 2:** Differences and similarities between two lung nodule types in *Pomacea canaliculata*

***Noduledescription***	***A. cantonensis-induced nodules***	***Newly discovered nodules***
Parasite	Allnodules containcurled larvae	No parasites inside
Size	Mostly similar in size:0.25±0.02mm	Different sizesranging from 0.1 to 2mm
Shape	Small spherical, with no transparent surrounding material	Poached-egg shape, with transparent material surrounding it
Color	Dark and bright regions	Even colored
Location	Lung	Lung
No. Positive snails/total number(rate)	4/447(0.9%)	14/447(3%)

### Prevalence of the two nodule types in *P. canaliculata*

Among the 447 *P. canaliculata* snails collected, four were *A. cantonensis*-infected (0.9% infection rate), showing *A. cantonensis*-induced nodules in the lung sacs, and 14 contained the new nodules (3% infection rate). The new nodules’ prevalence was much higher than that of the *A. cantonensis*-induced nodules.

### Microbial community composition in the new nodules

**Fig. 2:**
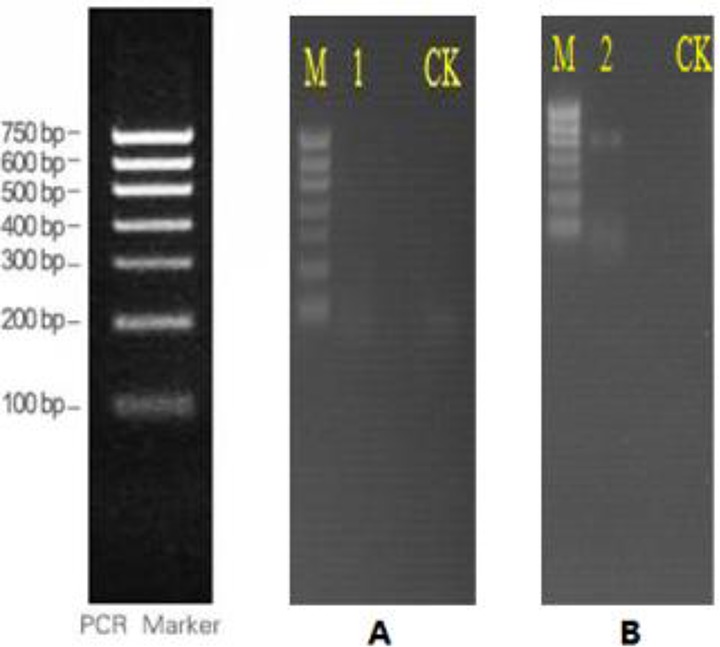
PCR results. A: Amplified by primer 1; B: Amplified by primer 2

The bacterial 16S rRNA gene of the newly discovered nodules was amplified by PCR, and the results exhibited no positive bands on electrophoresis (Fig. 2A), indicating that the new nodules might not be caused by bacterial infection. Conversely, the new nodules’ eukaryotic 18S rRNA gene was successfully amplified, which suggested these nodules were eukaryotic (Fig. 2B).

### Overall sequence descriptions

Overall, 43491 raw sequences were obtained from the new nodules, 40959(94.18%) of which ranged from 351–400bp (base pairs).

**Fig. 3:**
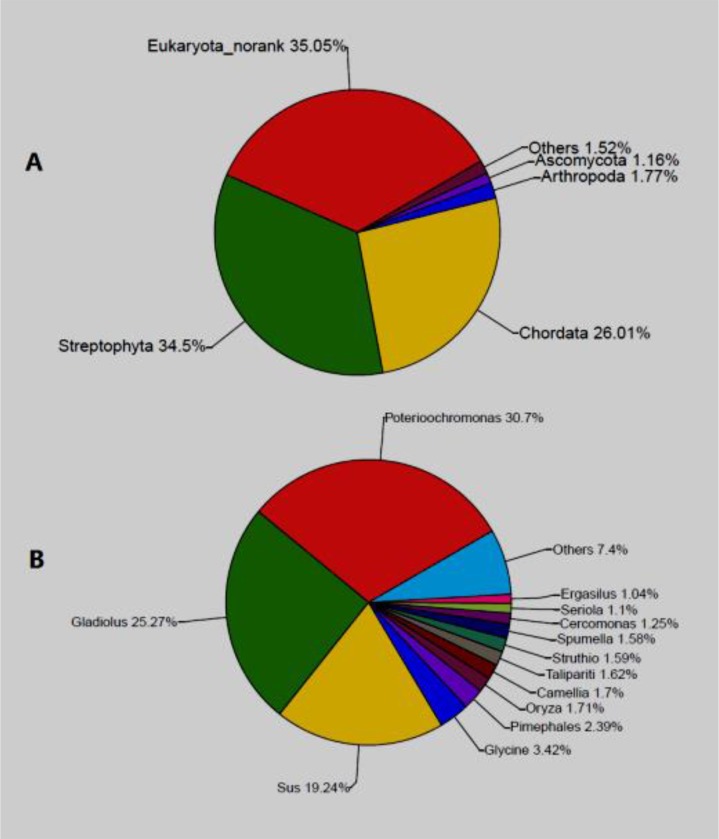
Community compositions of the new nodules. A: at phylum level; B: at genus level

Per the OTU analysis, the compositions of the new nodules were as follows. The dominant phyla were *Strepto phyta* (34.5%), Chor-data (26.01%), Arthropoda (1.77%) and Ascomycota (1.16%) (Fig. 3A). The dominant genera were Poterioochromonas (30.7%) and Gladiolus (25.27%) (Fig. 3B), and at the species level, *Poterioochromonas* sp. might be the causative pathological agent of the new nodules.

## Discussion

Nodules in snail hosts are common pathological structures (20, 21). In *Biomphalaria glabrata*, a novel bacterial pathogen-induced pathological nodules, widely distributed in the snail’s mantle, hepato-pancreas, and ovotestis, leading to increased mortality (22). In *Bulinus jousseaumei*, another bacterial pathogen caused nodules in superficial areas of the snail’s body, such as the pseudo branch, foot, collar, mantle, and tentacles (23). However, this has rarely been reported in *P. canaliculata* snails.

In addition, since *P. canaliculata* widely inhabit the freshwater system in tropical and sub-tropical zones where microorganisms are rich, this snail often forms facultative and obligate symbiotic associations with bacteria or viruses (24, 25), even other small invertebrates, such as *Temnocephala iheringi* (26), leeches of *Helobdella ampullarisae,* etc. (27, 28). Notably, two algae, *Climacodium frauenfeldianum* (29) and *Rhopalodia gibba* (30), were found in the *P. canaliculata*; however, no pathological changes similar to the new nodules were reported.

Algae *Poterioochromonas* sp. belongs to the family Dinobryonaceae, order Chromulinales, class Chrysophyeeae, and phylum Chrysophyta. This algae is widespread in freshwater (31, 32), sharing the similar environment with *P. canaliculata* snail. No previous report indicated algae could infect *P. canaliculata*, and this research is the first likely report of *Poterioochromonas* infection in *P. canaliculata* snails.

High-throughput sequencing (HTS) can effectively explore microbial biodiversity in the environment and digestive systems (33, 34). Sequencing rRNA gene polymerase chain reaction amplicons (rRNA tags), such as 16S (35) and 18S rRNA(36), is currently used to investigate microbial biodiversity. In this research, 18S rRNA sequencing was successfully used and indicated that eukaryotic cells, most likely *Poterioochromonas* sp., caused the new nodules.

## Conclusion

Snail facultative and obligate symbiotic associations with microorganisms or other creatures are common in the field, as are pathological nodules caused by environmental bacteria or viruses. However, our study determined the new nodules were a new type of pathological change in snail lungs, which differ from the *A. cantonensis*-induced nodules as shown by morphological observation, histopathological sections, and 18S rRNA gene amplification. *Poterioochromonas* sp. infection or other eukaryotic cell infections may cause these nodules; however, direct evidence must be confirmed by pathogenic infection experiments to ensure this. Moreover, 18S rRNA gene sequencing provides a powerful tool for studying micro-biodiversity in these nodules, but thus far, only limited knowledge of these nodules exists. The interconnection between the new nodules and the snail host, its physiological impact on the snail, and the new nodules’ formation require further study.
